# Elucidation of the Role of SHMT2 in L-Serine Homeostasis in Hypoxic Hepa1-6 Cells

**DOI:** 10.3390/ijms252111786

**Published:** 2024-11-02

**Authors:** Shuo Zhang, Ruoxu He, Mingsi Zhang, Jingcheng Zhang, Mengting Wu, Guangji Zhang, Tao Jiang

**Affiliations:** 1 School of Basic Medical Sciences, Zhejiang Chinese Medical University, Hangzhou 310053, China; zs15156020951@163.com (S.Z.); 20211009@zcmu.edu.cn (R.H.); 15328236888@163.com (J.Z.); wmt_0917@163.com (M.W.); 2 Musculoskeletal Sport Science and Health, Loughborough University, Loughborough LE11 3TU, UK; m.zhang4-23@student.lboro.ac.uk; 3 Zhejiang Key Laboratory of Blood-Stasis-Toxin Syndrome, Zhejiang Chinese Medical University, Hangzhou 310053, China

**Keywords:** L-serine metabolism, hypoxia, hepatocellular carcinoma, *SHMT2*, glycolysis

## Abstract

Hypoxia is a characteristic feature of malignancy; however, its effect on metabolism remains unclear. In this study, Hepa1-6 cells were cultured under hypoxic conditions and their metabolites were analyzed. Elevated levels of L-serine along with increased glycolytic activity are prominent features of hypoxia. Transcriptome sequencing revealed the downregulation of genes involved in L-serine synthesis and metabolism, which was confirmed by PCR analysis and comparison with public databases. Further experimental evidence indicates that the accumulation of L-serine under hypoxic conditions is attributable not only to enhanced glycolysis but also to a reduction in the catabolism of L-serine mediated by serine hydroxymethyltransferase 2 (*SHMT2*).

## 1. Introduction

Hypoxia is a hallmark of malignant tumors [1,2,3,4]. Unlike normal cells, tumor cells can adapt to a hypoxic environment [5,6,7]. These cells demonstrate a notable ability to thrive under hypoxic conditions, and exhibit aberrant behaviors, such as altered gene transcription, protein synthesis, and metabolic processes. Metabolism plays a crucial role in determining the fate of cells, as it supplies necessary nutrients and energy while also regulating cellular functions. In the absence of oxygen, tumor cells often adapt to survive by increasing anaerobic glycolysis [8,9]. Moreover, despite adequate oxygen availability, tumor cells prefer energy production via glycolysis, a phenomenon commonly referred to as the ‘Warburg effect’ [10,11,12]. Furthermore, tumor cells exhibit increased uptake of amino acids, particularly glutamine [13,14,15]. The metabolic traits of various cellular survival mechanisms, other than the anaerobic features mentioned earlier, remain unclear.

L-serine is a nonessential amino acid in the human body involved in protein synthesis and energy production. Elevated L-serine levels in tumors, such as hepatocellular carcinoma, contribute to tumorigenesis and are associated with clinical prognosis [16,17]. The maintenance of L-serine homeostasis and equilibrium is regulated by de novo synthesis, exogenous uptake, and catabolism. L-serine is a major product of glycolysis. After increased glycolysis, 3-phosphoglycerate (*3-PG*), a glycolytic derivative, is gradually converted to L-serine through a series of enzymatic reactions involving phosphoglycerate dehydrogenase (*PHGDH*), phosphoserine aminotransferase (*PSAT*), and phosphoserine phosphatase (*PSPH*) [18]. L-serine plays a crucial role in cancer cell metabolism. Studies have shown that tumor cells often exhibit a high demand for L-serine, leading to the upregulation of L-serine metabolism to support their rapid growth. Additionally, tumor cells frequently reprogram metabolic pathways to enhance L-serine synthesis and utilization, thereby adapting to hypoxic or nutrient-deprived environments [19,20]. Furthermore, L-serine metabolism is intertwined with other key metabolic pathways such as one-carbon metabolism, glycolysis, and lipid metabolism, affecting the metabolic network and adaptability of tumor cells [21,22].

In this study, we focused on L-serine metabolism in tumor cells under hypoxic conditions. We cultured Hepa1-6 cells under hypoxic conditions and conducted metabolomic and transcriptomic analyses to characterize the key metabolites and metabolic genes. Our findings indicate a significant increase in L-serine accumulation following hypoxia. The increase in L-serine pool accumulation is attributed not only to enhanced glycolytic flux but also to a reduction in L-serine catabolism mediated by *SHMT2* following hypoxia.

## 2. Results

### 2.1. Untargeted Metabolomics Analysis Reveals Abnormally Elevated L-Serine Levels Under Hypoxia

**Figure 1:**
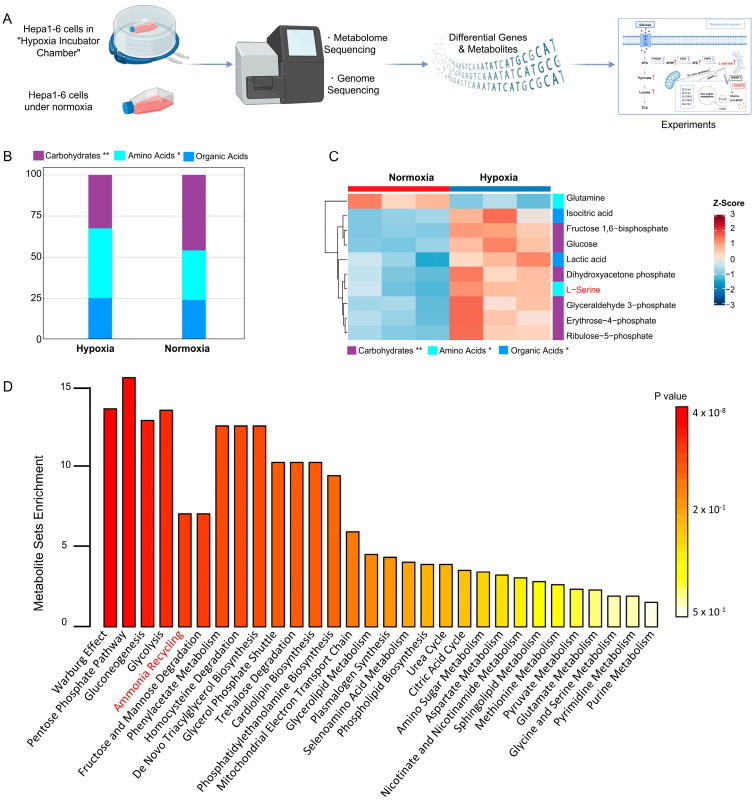
(**A**) Flowchart design of this study. (**B**) Composition of three major differential metabolites in Hepa1-6 cells under hypoxic and normoxic conditions: carbohydrates, amino acids, and organic acids. (**C**) Differentially abundant metabolites (top ten) under hypoxic and normoxic conditions. (**D**) Differentially abundant metabolite enrichment plot. * *p* < 0.05, ** *p* < 0.01.

To explore the effects of hypoxia on metabolism and gene regulation, we performed a series of experiments, as shown in the flow chart depicted in Figure 1A. Hepa1-6 cells were cultured under normoxic and hypoxic conditions. Metabolomic analysis of cells under these different conditions was performed using liquid chromatography–mass spectrometry (LC–MS), followed by the identification of key metabolites and associated metabolic genes through screening. The obtained high-throughput data were validated through a series of downstream experiments. In untargeted metabolomic analysis, we focused on three distinct metabolite groups: amino acids, carbohydrates, and organic acids. Following hypoxia, amino acid levels increased, carbohydrate levels decreased, and no significant changes in organic acids were observed (Figure 1B). Subsequent analysis of specific metabolites revealed the top 10 differentially abundant metabolites, as shown in the heatmap (Figure 1C). The levels of products associated with glycolysis, such as glucose, 1-6 glucose, and lactate, were notably elevated. Additionally, our findings indicated a notable reduction in glutamine levels and a substantial increase in L-serine levels, suggesting a profound impact on amino acid metabolism under hypoxic conditions. Further analysis revealed a significant enrichment of pathways related to the Warburg effect, glycogen synthesis, and glycolysis among the differentially abundant metabolites (Figure 1D). In particular, the results demonstrated significant enrichment of ammonia recycling pathways (Figure 1D). Previous studies have highlighted the interplay between cellular ammonia metabolism and amino acids, particularly L-serine. These findings suggest that elevated L-serine levels may be involved in the response of Hepa1-6 cells to hypoxia.

### 2.2. Transcriptome Sequencing Reveals L-Serine Metabolic Modules Repressed Under Hypoxia

**Figure 2:**
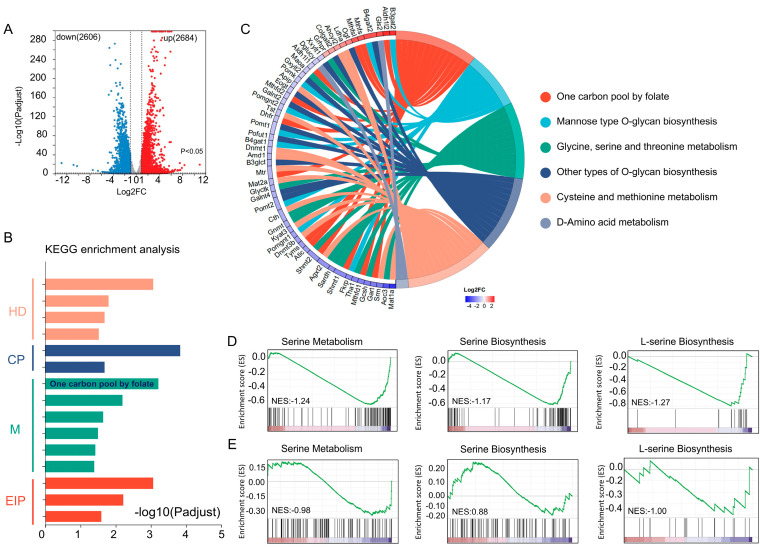
(**A**) Differentially expressed genes under hypoxia and normoxia (|log2FC| > 1, *p* < 0.05). (**B**) KEGG pathway enrichment plots of the differentially expressed genes. Note: human diseases (HDs), cellular processes (CPs), metabolism (M), environmental information processing (EIP). (**C**) Chordal plots of amino acid metabolism-related modules. (**D**) GSEA plots of the genes related to L-serine anabolism and GSEA plots from the GSE15505 dataset (**E**).

To identify the genes associated with metabolism in response to hypoxia, we conducted RNA-seq analysis of cellular genome expression. Figure 2A shows significant gene alterations in cells exposed to hypoxic conditions. We used an absolute log2FC greater than 1 and a *p*-value of less than 0.05 as the criteria for selecting differential genes, resulting in 2684 upregulated genes and 2606 downregulated genes. Subsequently, we examined these differentially expressed genes across four distinct categories: human diseases (HDs), cellular processes (CPs), metabolism (M), and environmental information processing (EIP) (Figure 2B). Our study focused on cellular metabolism. Specifically, the one-carbon pool was enriched with folate. One-carbon metabolism consists of folate and methionine cycles, with L-serine being identified as the primary supplier of one-carbon units [23,24,25]. These findings suggest that L-serine metabolism is affected by hypoxia. To investigate L-serine metabolism under hypoxic conditions, we examined the key modules of L-serine metabolism, and the results revealed the downregulation of most genes within these modules (Figure 2C). Additionally, we conducted GSEA on key gene sets related to L-serine metabolism, synthesis, and transformation. These findings revealed a general decrease in L-serine metabolism, including synthesis and uptake, as shown in Figure 2D. This observation was confirmed in Hep3B cells (Figure 2E). According to the metabolomics data in conjunction with these results, L-serine plays a critical role in hepatocellular carcinoma cells under hypoxic conditions.

### 2.3. Highly Abundant L-Serine Pools Under Hypoxia Are Not Completely Regulated by Synthesis and Uptake

**Figure 3:**
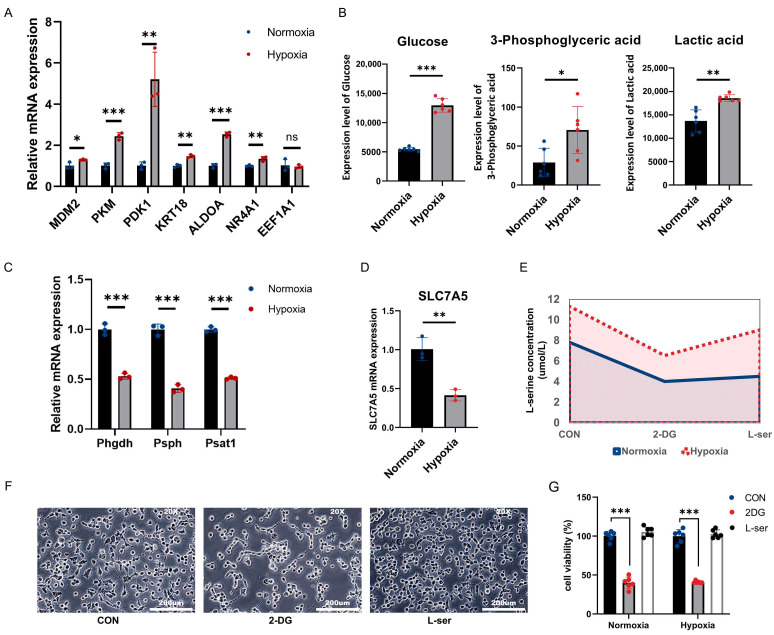
(**A**) mRNA expression levels of glycolysis-related genes. (**B**) Levels of glycolysis-related products. (**C**) Expression levels of key genes in the L-serine biosynthesis pathway. (**D**) mRNA expression levels of the L-serine uptake gene *SLC7A5*. (**E**) Levels of intracellular L-serine in cells cultured with 2-DG or L-serine restriction. (**F**) Morphology of Hepa1-6 cells under conditions of 2-DG addition and L-serine deprivation (20×). (**G**) Proliferation of Hepa1-6 Cells under conditions of 2-DG addition and L-serine deprivation. ns *p* > 0.05; * *p* < 0.05, ** *p* < 0.01, *** *p* < 0.001.

To further clarify the cause of elevated L-serine levels, we first examined the expression of glycolysis-related genes, which showed significantly increased expression in tumor cells under hypoxia. We found that most glycolysis-related genes, except for eukaryotic translation elongation factor 1 alpha 1 (*EEF1A1*), were significantly upregulated after hypoxia (Figure 3A). Key metabolite analysis further demonstrated increased glycolysis under hypoxia (Figure 3B), which was consistent with previous studies. One of the major sources of L-serine is glycolysis-related synthesis and exogenous uptake, and we hypothesized that the elevation of L-serine levels was due to either elevated glycolysis or increased uptake. We examined key genes involved in L-serine synthesis and detected a significant decrease in the expression of *PHGDH*, *PSPH*, and phosphoserine aminotransferase 1 (*PSAT1*), which was consistent with the GSEA results (Figure 3C). Similarly, expression of the key gene involved in L-serine uptake, solute carrier family 7 member 5 (*SLC7A5*), was significantly downregulated (Figure 3D). This suggests that elevated L-serine levels are not caused exclusively by L-serine synthesis and uptake. To further support these findings, we used 2-Deoxy-D-glucose (2-DG) to inhibit glycolytic flux and exogenous L-serine restriction to reduce L-serine uptake. Neither treatment completely blocked the increase in the L-serine pool under hypoxia; in particular, there was no significant change in L-serine restriction under hypoxia (Figure 3E). Cell proliferation was observed under these conditions. The results showed that 2-DG significantly inhibited cell proliferation under hypoxia, which was consistent with the results of a large number of studies [26]. However, the change in exogenous L-serine did not affect cell proliferation (Figure 3F). Overall, we conclude that the increase in L-serine under hypoxia is partly caused by elevated glycolysis but has little effect on exogenous L-serine uptake.

### 2.4. Reduced SHMT2-Mediated Catabolism Is Involved in the Stabilization of the L-Serine Pool Under Hypoxia

**Figure 4:**
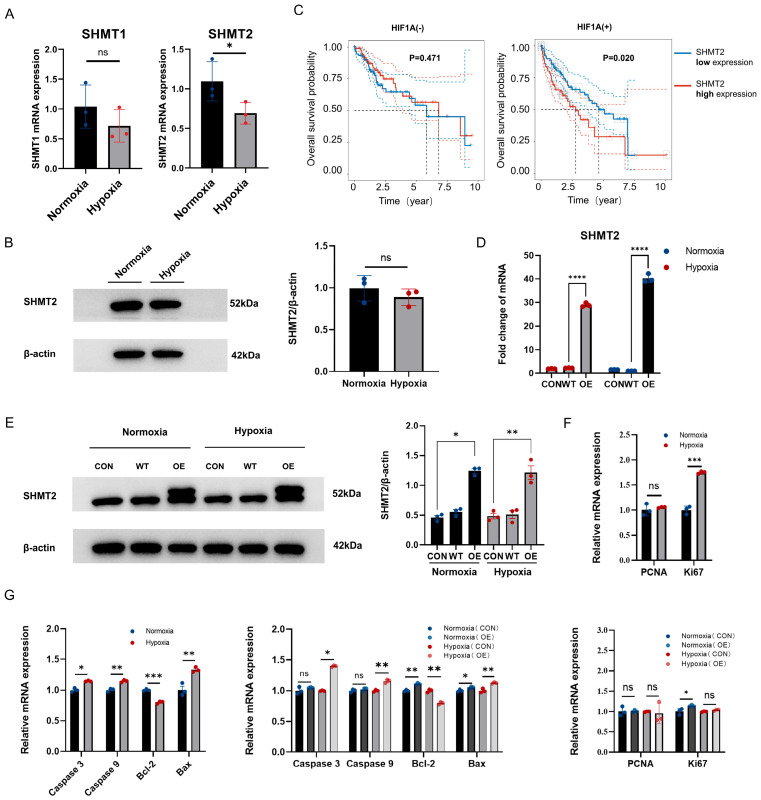
(**A**) mRNA expression levels of the *SHMT1* and *SHMT2* genes. (**B**) Expression level of the SHMT2 protein. (**C**) Prognostic analysis of high and low *SHMT2* expression in HIF1A-positive and negative patients (TCGA data). (**D**) Evaluation of *SHMT2* overexpression efficiency (OE) at the genetic level. (**E**) Assessment of SHMT2 overexpression efficiency (OE) at the protein level. (**F**) Expression levels of molecular markers directly associated with cell proliferation processes. (**G**) Expression levels of core members of the apoptosis pathway family. ns *p* > 0.05; * *p* < 0.05, ** *p* < 0.01, *** *p* < 0.001, **** *p* < 0.0001.

**Figure 5:**
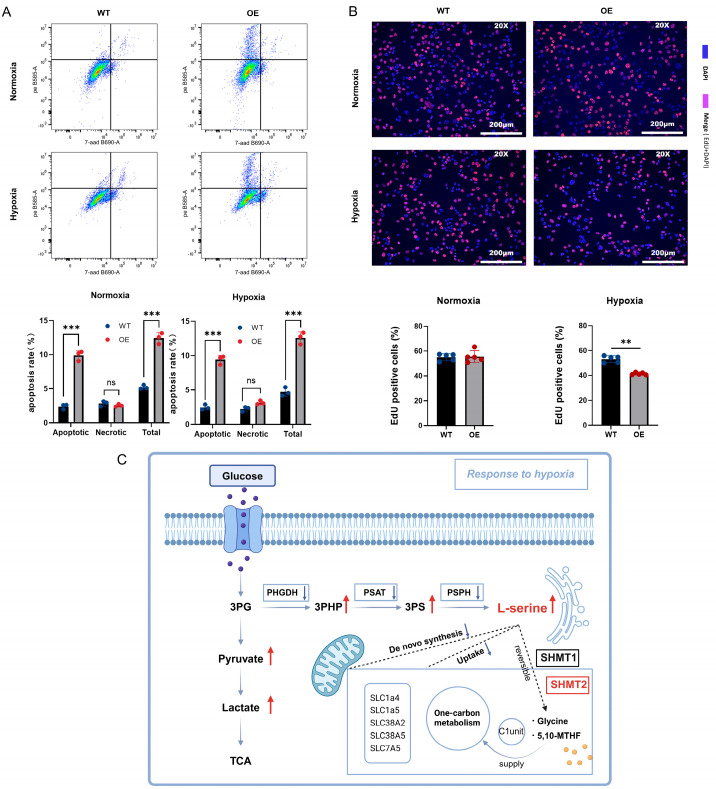
(**A**) Analysis of apoptosis in Hepa1-6 cells under normoxic and hypoxic conditions using flow cytometry. (**B**) Detection of cell proliferation in Hepa1-6 cells under normoxic and hypoxic conditions using EdU staining. (**C**) High L-serine response of Hepa1-6 cells under hypoxia: Hypoxia leads to increased glycolysis, increased 3PHP and 3PS, and elevated L-serine production. A high L-serine pool causes the downregulation of amino acid synthesis genes such as *PHGDH*, *PSAT1*, and *PSPH*, which inhibit the de novo synthesis of L-serine, as well as the downregulation of L-serine uptake genes such as *SLC7A5*, which inhibits the exogenous uptake of L-serine. Additionally, downregulating *SHMT2* inhibits the catabolism of L-serine into glycine and *5,10-MTHF*. Together, these factors maintain high levels of L-serine stabilization in response to cellular metabolism under hypoxia. ns *p* > 0.05; ** *p* < 0.01, *** *p* < 0.001.

In addition to L-serine synthesis and exogenous uptake by the glycolytic pathway, the catabolism of L-serine within cells contributes to the regulation of the L-serine pool. Serine hydroxymethyltransferase (*SHMT*) is a key component of L-serine catabolism that converts L-serine to glycine while generating 5,10-methylenetetrahydrofolate (*5,10-MTHF*). SHMT exists in two isoforms: *SHMT1* and *SHMT2* [27,28]. We hypothesized that highly abundant L-serine pools are associated with reduced L-serine catabolism. This result was experimentally demonstrated by the fact that hypoxia reduced the expression of *SHMT1* and *SHMT2*, with more pronounced changes in *SHMT2* (Figure 4A). At the protein level, the SHMT2 expression was consistent with previous findings. However, under hypoxic conditions, the downregulation of SHMT2 protein was not significant (Figure 4B). In addition, in the TCGA database, *SHMT2* had prognostic value in HIF1A-positive patients but not in HIF1A-negative patients, suggesting that *SHMT2* was more strongly associated with hypoxia (Figure 4C). These findings suggest that reduced L-serine catabolism is involved in maintaining highly abundant L-serine pools in hepatocellular carcinoma cells. We overexpressed *SHMT2* using a plasmid to promote L-serine catabolism (Figure 4D,E). Subsequently, molecular markers directly associated with cell proliferation processes, including proliferating cell nuclear antigen (*PCNA*) and the marker of proliferation Ki-67 (*Ki67*) [29,30], as well as core members of the apoptosis pathway families, such as the caspase family and Bcl2 apoptosis regulator (*Bcl-2*) family [31,32,33], were assessed. The results revealed significant differences between hallmark molecules under normoxic and hypoxic conditions. Following *SHMT2* overexpression, pro-apoptotic molecules showed varying degrees of elevation (Figure 4F,G). We believe that elevated *SHMT2* levels promote L-serine catabolism and decrease L-serine pool. In particular, when *SHMT2* expression was elevated, apoptosis, especially early apoptosis, was significantly promoted in Hepa1-6 cells under normoxic and hypoxic conditions (Figure 5A). Overexpression of *SHMT2* significantly affected cell proliferation under hypoxia but had no effect under normoxia (Figure 5B). These results suggest that the stabilization of the L-serine pool is important for cellular homeostasis, especially under hypoxic conditions. In hepatocellular carcinoma cells, an elevated L-serine pool was significantly correlated with *SHMT2*-mediated L-serine catabolism and elevated glycolytic flux (Figure 5C).

## 3. Discussion

Metabolic alterations in tumor cells are important factors that influence the malignant behavior of tumors. The rapid proliferation of tumors and angiogenesis lead to internal heterogeneity and the presence of large areas of hypoxia. How tumor cells respond to metabolism under hypoxic conditions is an important question that needs to be explored. In this study, we used Hepa1-6 cells as a model to investigate the effects of hypoxia on HCC cell metabolism. The key metabolites and related genes were identified using untargeted metabolomics and transcriptomics. Our results suggest that elevated L-serine levels are a key metabolic response to hypoxia. Furthermore, our results suggest that the elevated L-serine pool is not solely caused by elevated glycolytic flux but is also associated with reduced catabolism of L-serine mediated by *SHMT2*, which is important for maintaining the stability of the L-serine pool and cell proliferation under hypoxia.

Hypoxia is an important factor in malignant tumors and metabolic reprogramming under hypoxia is an important feature of malignant tumors. The ability of tumor cells to respond to hypoxic stress is closely related to adaptive metabolic alterations. Numerous studies have shown that the glycolytic flux is increased in tumor cells in response to hypoxia. Therefore, most studies have focused on the regulation of glycolysis. However, determining how to target glycolysis to curb tumor progression requires a collaborative approach of many researchers [34]. An in-depth investigation of metabolism under hypoxic conditions is the basis for the development of metabolism-related tumor therapeutics. L-serine is closely related to the proliferation of tumor cells, which require large amounts of L-serine for growth and proliferation. Deprivation of L-serine from tumor cells can substantially reduce their proliferative capacity [35,36]. L-serine is the predominant form of L-serine with biological activity and is involved in numerous cellular processes [37]. However, because L-serine is an important branch of glycolysis that can be synthesized under hypoxia, this key metabolic participant is often overlooked. Similarly, our results revealed a significant increase in L-serine levels after hypoxia. During glycolysis, 3-phosphoglycerate (*3PG*) is converted to 3-phosphohydroxypyruvate (*3PHP*) by *PHGDH*. *PSAT1* catalyzes the conversion of 3PHP to 3-phosphoserine (*3PSer*). Finally, *3PSer* is catalyzed by *PSPH* to produce serine, which is essential for the maintenance of the cellular one-carbon cycle and coordinated stabilization of ammonia. Our results are consistent with the fact that increased glycolysis after hypoxia leads to the accumulation of L-serine. Interestingly, this result suggests a diminished ability to synthesize and take up L-serine, suggesting that tumor cells deliberately regulate and maintain reasonable levels of L-serine. In addition, L-serine catabolism was significantly regulated by hypoxia. Normally, high levels of L-serine are regulated and processed through various pathways, including its conversion to glycine via *SHMT*, transformation into pyruvate by L-serine dehydratase, involvement in phospholipid and protein synthesis, and entry into the gluconeogenesis pathway to maintain homeostasis [22]. However, tumor cells decrease L-serine catabolism to maintain high levels of the L-serine pool. We targeted two key *SHMT* enzymes, *SHMT1* and *SHMT2*, which play important roles in the conversion of L-serine and glycine in the cytoplasmic one-carbon pathway and catalyze L-serine from the cytosol and mitochondria, respectively, to maintain L-serine levels [38]. Here, we found that *SHMT2* is more strongly regulated by hypoxia than is *SHMT1*. Reduced *SHMT2* levels decreased the conversion of L-serine to glycine, favoring high levels of L-serine. Notably, however, *SHMT1* has been more often studied as an oncogene in some tumors, and we found the opposite results. High levels of *SHMT2* promote L-serine catabolism, resulting in the attenuated proliferation and accelerated death of tumor cells under hypoxia. This result also provides some unique insights, as genes may play different roles under hypoxia and normoxia, which could also be one of the reasons some drugs are ineffective or generate resistance in the treatment of tumors.

Our study had some limitations. These limitations include the small sample size for sequencing and failure to validate this phenomenon in a variety of other cell lines. Moreover, how the synthesis, exogenous uptake, and catabolic transport of L-serine are directly and dynamically regulated in the glycolytic pathway needs to be analyzed in detail. Most importantly, the effects of high levels of L-serine on tumor proliferation under hypoxic conditions have not been elucidated. However, we found that L-serine is a regulatory manifestation of hepatocellular carcinoma in response to hypoxia and may be a target in the field of tumor metabolism.

## 4. Materials and Methods

### 4.1. Cell Culture

The murine liver cancer cell line Hepa1-6 used in this study was provided by the Zhejiang Academy of Traditional Chinese Medicine (Hangzhou, China). The cells were cultured in DMEM (Procell, Wuhan, China) supplemented with 10% fetal bovine serum, penicillin (100 U/mL), and streptomycin (0.1 mg/mL) and kept in a humidified incubator at 37 °C and 5% CO_2_. For hypoxic culture, the cells were placed in hypoxic chambers (Billups-Rothenberg, San Diego, CA, USA) with 93% N_2_, 5% CO_2_, and 2% O_2_.

### 4.2. Nontargeted Metabolomics Sequencing

Under normoxic and hypoxic conditions, Hepa1-6 cells were digested with trypsin, resuspended in phosphate-buffered saline (PBS), and counted. The same number of cells (1 × 10^7^ cells) were placed into new 1.5 mL centrifuge tubes. The samples were centrifuged at 500× *g*; the supernatant was discarded and any residual liquid was thoroughly aspirated. Nontargeted metabolomic assays were performed using the XporeMET platform (Metabo Profile, Shanghai, China).

### 4.3. Transcriptomic Sequencing

The sample processing steps for Hepa1-6 cells are described in Section 4.2. Cells cultured under various conditions were collected and subjected to RNA extraction using the TRIzol reagent, followed by library sequencing using the Illumina NovaSeq Reagent Kit (Illumina, San Diego, CA, USA). The obtained data were processed using FASTP for quality control, HISAT2 for alignment with reference genes, and RSEM for expression analysis. Subsequent data analyses, including differential expression analysis with DESeq2, Gene Ontology (GO), Kyoto Encyclopedia of Genes and Genomes (KEGG) pathway analysis, and GSEA, were conducted using the Meggie Cloud platform. Specifically, in the KEGG pathway enrichment analysis, we selected the top 15 pathways and categorized them based on their biological processes: human diseases (HDs), cellular processes (CPs), metabolism (M), and environmental information processing (EIP). Additionally, we used Gene Set Enrichment Analysis (GSEA) to determine whether the predefined gene set (such as L-serine biosynthesis) exhibits statistical differences between normoxia and hypoxia. We ranked the genes of interest based on their differential expression levels in the two sample groups to create a gene set for analysis. By comparing the positions of genes from the predefined gene set within the ranked gene set, we can assess whether the pathway is upregulated or downregulated. Each black vertical line represents a gene from the predefined gene set and its corresponding position in the ranked gene set. The vertical axis, Enrichment Score (ES), indicates the enrichment level of genes from the predefined gene set within the ranked gene set. The Normalized Enrichment Score (NES) reflects the standardized enrichment score. When the score reaches its highest peak or lowest trough, it is used as the enrichment score for the predefined gene set; the greater the absolute value of the NES, the higher the significance.

### 4.4. Acquisition and Analysis of GEO Data

The sequencing data for GSE155505, including hypoxic and normoxic culture data for the six Hep3b samples, were retrieved from the GEO database. The key signaling pathways associated with L-serine were analyzed using GSEA v3.0, as outlined in the literature.

### 4.5. Plasmid Transfection

The *SHMT2* overexpression plasmid was synthesized by Jiman Biotechnology Co (Shanghai, China), and contained the *SHMT2* gene with a length of 1437 bp, transcript number NM_009171.2, and the PGMLV-6751 vector. Hepa1-6 cells were transfected with the constructed overexpression plasmid after reaching 70–80% confluency in Petri dishes following appropriate passages. Two hours prior to transfection, the cells designated for transfection were replenished with fresh DMEM (Thermo Fisher Scientific, Waltham, MA, USA). Transfection was performed according to the manufacturer’s instructions and the prepared nucleic acid–transfection reagent complex was carefully added dropwise to the culture wells. The plate was gently shaken in a horizontal manner to ensure uniform mixing of the components before being placed in a humidified incubator set at 37 °C with 5% CO_2_ for incubation. After a 4 h incubation period, the medium was replaced, and the cells were further incubated for an additional 24 h.

### 4.6. Detection of L-Serine

Quantitative analysis of intracellular L-serine was conducted using a mouse L-serine quantitative detection kit (zcibio, Shanghai, China). Standard and sample wells were arranged in accordance with the manufacturer’s guidelines, followed by sequential steps, including sampling, the addition of horseradish peroxidase (HRP), washing, the addition of substrate, and the addition of termination solution. Absorbance readings were taken at 450 nm and data analysis was performed using the ELISA Calc regression fitting calculation program v.0.1. The resulting four-parameter logistic curve fit of the standards exhibited an r^2^ value exceeding 0.99.

### 4.7. CCK-8 Assay

Cell viability was assessed using the Cell Counting Kit-8 (Beyotime, Shanghai, China) assay by inoculating 1.5 × 10^4^ cells into the wells of a 96-well plate. Following a 6-h incubation under normal oxygen and hypoxic conditions, DMEM was replaced with medium containing 2-DG (MCE, Dallas, TX, USA) or L-serine for an additional 18 h of incubation. Subsequently, the medium in each well was replaced with DMEM containing a 1% concentration of CCK-8 reagent and the cells were incubated in a humidified incubator at 37 °C and 5% CO_2_ for 0.5 h in the absence of light. The absorbance was measured at 450 nm using an enzyme label (BioTek, Winooski, VT, USA).

### 4.8. PCR

Total RNA was extracted using an RNA extraction kit (Vazyme, Nanjing, China), according to the manufacturer’s guidelines. Subsequently, the RNA concentration was assessed using an ultra micro spectrophotometer (Thermo Fisher Scientific, Waltham, MA, USA). Total RNA (1 µg) from each sample was used for cDNA synthesis. Reverse transcription was conducted for 40 min in a PCR thermocycler (Bio-Rad, Hercules, CA, USA) using a premixed solution for real-time quantitative PCR amplification and SYBR Green Master Mix (Yeasen, Shanghai, China). Primers were synthesized by Jin Wei Zhi Biologicals (Shanghai, China). The primer sequences are listed in Table 1.

### 4.9. Western Blot

SHMT2-overexpressing plasmid-transfected and non-transfected cells were cultured for 24 h under normoxic and hypoxic conditions. Cells were lysed with precooled RIPA buffer (Beyotime, Shanghai, China) and a protease inhibitor (Beyotime, Shanghai, China) for 20 min. Centrifugation was performed at 12,000 rpm for 20 min and the supernatant was collected to measure protein concentration. Equal amounts of total protein were separated by SDS-PAGE (Boster, Wuhan, China) and transferred to PVDF membranes (Merck Millipore, Burlington, MA, USA), which were blocked with skim milk powder (Yuan Ye, Hangzhou, China) for 1 h. The membranes were incubated with SHMT2 primary antibody (CST, New York, NY, USA) at 4 °C overnight. After washing with TBST, the membranes were incubated with corresponding peroxidase-conjugated secondary antibodies for 1 h at 37 °C. Development was performed using an enhanced chemiluminescence reagent (FD, Hangzhou, China).

### 4.10. EdU Staining

EdU (5-ethynyl-2′-deoxyuridine) staining is a technique used to detect cell proliferation. During the S phase of the cell cycle, EdU is incorporated into newly synthesized DNA, replacing thymidine (dTTP). The incorporated EdU can then be conjugated to a fluorescent dye through a click chemistry reaction, creating a detectable fluorescent signal. This method allows for the identification and quantification of cells that have undergone DNA synthesis using fluorescence microscopy. Cell proliferation following transfection with an *SHMT2* overexpression plasmid was evaluated using the EdU Cell Proliferation Imaging Analysis Kit (Abbkine, Wuhan, China). Specifically, cells were seeded in 96-well plates and treated with EdU (10 µM) for 1 h. Subsequently, the cells were fixed with PBS buffer containing 3.7% formaldehyde and permeabilized with PBS buffer containing 0.5% Triton X-100. The Click-iT reaction mixture was prepared according to the manufacturer’s instructions, incubated at room temperature in the absence of light, and analyzed for labeled DNA using fluorescence microscopy.

### 4.11. Flow Cytometry

The PE Annexin V and 7-AAD dual staining assay for detecting apoptosis is based on phosphatidylserine (PS) externalization and changes in cell membrane integrity. PE Annexin V binds to PS, which flips to the outer membrane in early apoptotic cells, while 7-AAD can only penetrate cells with compromised membranes, such as late apoptotic or necrotic cells, where it binds to DNA. Through flow cytometry, this double-staining method can detect apoptosis. In flow cytometry, the 585 and 690 refer to optical channels that detect fluorescence signals emitted by specific dyes within defined wavelength ranges. The 585 channel is typically used to detect the fluorescence of PE Annexin V, while the 690 channel is used to detect the fluorescence of 7-AAD. A PE Annexin V Apoptosis Detection Kit I kit (BD, Franklin Lakes, NJ, USA) was used in accordance with the manufacturer’s instructions. The cells were prepared as a single-cell suspension (1 mL) at a concentration of 1 × 10^6^ cells/mL, washed with precooled PBS, and resuspended in 100 mL binding buffer. Subsequently, 5 µL of PE membrane-associated protein V and 7-AAD were added and incubated at room temperature in the absence of light for 15 min, followed by flow cytometry analysis within 1 h. The samples were assessed using flow cytometry and results were analyzed using Flow Jo 10.9.0.

### 4.12. Statistical Analysis

Each experiment was repeated at least thrice. The differences between the components were compared using the Student’s *t*-test. A *p*-value greater than 0.05 was considered to indicate no statistically significant difference (ns *p* > 0.05) while a *p*-value less than 0.05 was considered to indicate a statistically significant difference (* *p* < 0.05, ** *p* < 0.01, *** *p* < 0.001, **** *p* < 0.0001).

## 5. Conclusions

This study reveals the crucial role of L-serine in hepatocellular carcinoma cell metabolism under hypoxic conditions. Hypoxia leads to an increase in L-serine levels, which is not only attributed to enhanced glycolytic flux but also to a reduction in L-serine catabolism mediated by *SHMT2*. By increasing *SHMT2* expression to promote L-serine catabolism, L-serine pool homeostasis can be disrupted, effectively inducing apoptosis in tumor cells under hypoxic conditions. These findings suggest that targeting the L-serine metabolism may be a novel therapeutic strategy for cancer treatment.

## Figures and Tables

**Table 1:** Primers used for qRT–PCR analysis.

Gene	Primer Sequence (5′-3′)
*SLC7A5*	F: ATGGAGTGTGGCATTGGCTT
	R: GAGCACCGTCACAGAGAAGAT
*MDM2*	F: GTGAAGGGTCGGAAGATGCG
	R: GTTTTGGTCTAACCTGGAGGC
*PKM*	F: GCAGCGACTCGTCTTCACTT
	R: ATGGTTCCTGAAGTCCTCGG
*PDK1*	F: AGGATTACTTTATAGACCGGGTCAG
	R: TACGGATGGGGTCCTGAGAA
*KRT18*	F: ACTGGTCTCAGCAGATTGAGG
	R: CTCCGTGAGTGTGGTCTCAG
*ALDOA*	F: GCGTTCGCTCCTTAGTCCTT
	R: CATGGGTCACCTTGCCTGG
*NR4A1*	F: GACCAGGCATGGGCGAC
	R: CTGTCCATGGTGGCCTGG
*EEF1A1*	F: CGTCGTAATCGGACACGTAGA
	R: AGGAGCCCTTTCCCATCTCA
*SHMT1*	F: CCACCCTGTGGGCTTCTCAT
	R: TTCTCCGAGGCAATCAGCTC
*SHMT2*	F: CCCAAACTGGCCTCATCGAC
	R: TGTGCCCTGACCTCATCACA
*PCNA*	F: CAATTTCTAGCAACGCCTAAGAT
	R: AAGAGGAAGCTGTGTCCATAGAG
*ki67*	F: CGAAAGTTGCGAAACATGTC
	R: TGCTTGTGGGTTTCTTTGG
*Caspase-3*	F: TGACCATGGAGAACAACAAAACCT
	R: TCCGTACCAGAGCGAGATGACA
*Caspase-9*	F: CACAGCAAAGGAGCAGAGAG
	R: TCTGAGAACCTCTGGCTTGA
*Bcl-2*	F: ATGTGTGTGGAGAGCGTCAAC
	R: AGACAGCCAGGAGAAATCAAAC
*Bax*	F: TGGAGATGAACTGGACAGCA
	R: GATCAGCTCGGGCACTTTAG
*β-actin*	F: GATGACGATATCGCTGCGCTG
	R: GTACGACCAGAGGCATACAGG
*18s*	F: GGCCGTTCTTAGTTGGTGGAGCG
	R: CTGAACGCCACTTGTCCCTC

## Data Availability

The datasets presented in this study can be found in online repositories. The transcriptomic data can be found in the GSA database: https://ngdc.cncb.ac.cn/gsa/browse/CRA019736, CRA019736 (accessed on 18 October 2024). The metabolomic data can be found in the OMIX database: https://ngdc.cncb.ac.cn/omix/release/OMIX007649, OMIX007649 (accessed on 22 October 2024).
